# The Role of the Sensorimotor System in Cognitive Functions

**DOI:** 10.3390/brainsci12050604

**Published:** 2022-05-05

**Authors:** Laila Craighero

**Affiliations:** Department of Neuroscience and Rehabilitation, University of Ferrara, Via Fossato di Mortara 19, 44121 Ferrara, Italy; crh@unife.it

The discovery of neurons with sensory properties in frontal motor circuits, and the discovery that these circuits send modulatory signals to the sensory parietal areas, strongly challenged the classical idea of a motor system as a mere executor of commands, and suggested that the sensorimotor system may contribute to the cognitive processes necessary for interaction with the world. On this basis, embodied cognition theory states that the mind, body, and its surrounding environment are highly interrelated, and hence, mutually dependent upon each other. In this view, human cognition is deeply rooted in the body’s interactions with its physical environment. One key notion of embodiment is the sharing of neural resources between cognitive and sensorimotor processes. In this Special Issue, “The Role of the Sensorimotor System in Cognitive Functions”, belonging to the section “Behavioral Neuroscience” of *Brain Sciences*, a range of exciting contributions (eight articles and one review) provide evidence of the involvement of the sensorimotor system during tasks addressing different cognitive functions, such as perception of time, space coding, kinesthetic imagery, and processing of concrete and abstract words. Furthermore, findings are reported suggesting that this relationship is causal, and that specific sensorimotor training improves the related cognitive functions.

Castellotti et al. [1] and Petrizzo et al. [2] investigated the influence of concurrent motor tasks on time estimation. Time can be defined as a continuous sequence of events that occur from the past, through the present, to the future. Experimental studies measuring time estimation make it clear that the perceived duration of events differs significantly from person to person and that each person’s time perception is affected by multiple internal and contextual factors. Increasing body temperature leads to an underestimation of time. Increasing arousal lengthens the perceived duration of events, whereas its decrease shortens duration estimation. Other influencing factors are stress and anxiety, sleep, drugs intake, and biological variables, such as age and gender. Finally, time estimation accuracy could be influenced by experience in particular fields involving time counting, such as music or sport. Interestingly, fine arm movements execution and walking cause an expansion of the perceived duration of concurrent stimuli. Castellotti et al. [1] wanted to investigate how concurrent cognitive and motor tasks interfere with the estimation of longer durations than those normally studied (i.e., a few seconds). They requested that participants perform cognitive tasks of different difficulties (look, read, solve simple and hard mathematical operations) and estimate durations of up to two minutes, while walking or sitting. The results showed that if observers pay attention only to time without performing any other mental task, they tend to overestimate the durations. Meanwhile, the more difficult the concurrent task, the more they tend to underestimate the time. These distortions are even more pronounced when participants are walking. These findings indicate that cognitive and motor systems interact nonlinearly and interfere with time perception processes, suggesting that they all compete for the same resources. Petrizzo et al. [2] were interested in clarifying whether distortions of time are induced only during the execution of actions, or whether the distortion persists after completion of motor activity, when several physiological variables, such as heart rate, remain altered, relative to the baseline. Participants made a temporal comparison in three different conditions: at rest, during sustained physical exercise (running on a treadmill), or immediately after the exercise. In line with previous reports, perceived duration was expanded during the motor routines. Furthermore, time judgements were not distorted for stimuli presented soon after the end of the physical activity. This result indicates that the distortion of perceived duration is related to the movement itself and not to the other physiological variables that are changed during the activity, which take time to revert to baseline levels.

Not only time but also space coding is influenced by the involvement of the sensorimotor system. A large proportion of languages in the world make a fundamental binary distinction between terms that refer to something that is a short distance away and terms that refer to something that is a great distance away. Accordingly, the adverb of place "far" is used to indicate who or what is at a great distance, while the "near" one to indicate who or what is close to where one is.. Like the perception of time, that of space is also influenced by many factors, such as the energetic costs associated with performing distance-relevant actions, the observer’s purposes, and the behavioral abilities of the observer’s body. A series of neuropsychological, behavioral, and neurophysiological studies suggest that the binary cognitive/linguistic distinction of space into near and far is not defined by metrical parameters but by functional ones, that is, near space is the space in which objects can be acted upon and a clear interaction is present, and far space is the space in which objects can only be perceived. Craighero and Marini [3] investigated the not-yet-studied cognitive association between spatial adverbs and actions with different functional characteristics. In addition, they extended the research to digital space. Indeed, as with physical space, in digital space our behaviors may be divided into perceiving or acting. The terms used to categorize these different online behaviors are, respectively, “content consumption” and “content generation”. Content consumption refers to the act of reading, listening, viewing, and other ways of taking in various forms of digital media. Content generation, instead, describes the various practices that result in any type of digital content, including text and voice messages, video files, photos, etc., to be shared with the digital community via blogs, email apps, and social media sites. The second objective of this work was, therefore, to study for the first time the presence of implicit associations between spatial adverbs and app icons that direct to online actions with different functional characteristics. Participants were involved in an implicit association test (IAT), a research tool based on reaction time recordings for indirectly measuring the strength of associations among categories. As expected, results showed an association between near/grasp, and far/look at, in the physical environment, and between near/content generation apps, and far/content consumption apps, in the digital one. These findings suggest that the distinction in the use of proximal or distal space adverbs depends on the characteristics of the actions potentially suitable to be performed in that space, and that adverbs of space also apply to digital space. A further indication of the central role of potential actions in space coding was provided by Tosoni et al. [4]. They considered the potential affordance relationship between the spatial features of far space and locomotion. Participants were requested to execute a walking-related action (i.e., a footstep ahead) in response to repeated presentations of pictures of an environmental layout framed from a far/panoramic vs. near/restricted view with respect to the observer. Pictures were presented in pairs (prime and target) and the footstep action was executed at the onset of the target picture on the basis of the perceptual match with the prime picture. Consistently with the hypothesis, results showed a facilitation effect for the execution of a footstep action in response to pictures framing an environment from a far perspective. Furthermore, they investigated whether the effect was associated with a significant modulation of the neurophysiological activity during processing of the prime and target stimulus and the timing of these modulations. To this aim, a data-driven approach was employed to determine whether the EEG event-related potentials (ERPs) recorded during the prime target interval were modulated by the framing distance of the environmental layout. The findings indicated a stronger ERP in response to prime images framing the environment from a far vs. near distance, and an inversion of polarity for far vs. near conditions during the subsequent target period associated with spatially directed foot-related actions. In general, these findings reveal a preferential affordance relationship between the distant large-scale environment and locomotion.

Oldrati et al. [5] addressed the theme of imagination, the faculty that produces ideas and images in the absence of direct sensory data. Specifically, they considered motor imagery (MI), the mental simulation and subjective experience of movement in the absence of overt execution of the corresponding motor output. Consistent evidence suggests that motor imagery involves the activation of several sensorimotor areas also involved during action execution, including the dorsal premotor cortex (dPMC) and the primary somatosensory cortex (S1). In the light of the overlap between regions recruited during MI and actual movement execution, increasing attention has been devoted to exploring the application of MI tasks in rehabilitation settings for patients suffering from a significant decrease in functional mobility, as well as in training for professional athletes and musicians. However, MI can be performed by distinct modalities, with the two most common modalities engaging kinesthetic and visual sensory experiences. Kinesthetic MI (kMI) is a form of mental motor rehearsal focusing on how a movement “feels” in terms of perceptions deriving from our own body during the execution of the movement. Experimental instructions targeting kMI require participants to pay attention to the somatosensations that they would normally perceive during the execution of a movement, such as muscle stretching and contractions or tactile sensations. Visual MI (vMI), conversely, mainly involves the visualization of a movement that can be either achieved from a first-person perspective, also referred to as internal vMI (i.e., with the image viewed by the subject’s own eyes), or from a third-person perspective, also referred to as external vMI (i.e., with the image viewed by an external observer’s standpoint). The authors aimed to investigate whether the involvement of sensorimotor areas is specific for either kinesthetic or visual imagery, or whether they contribute to motor activation for both modalities. They combined 1 Hz repetitive transcranial magnetic stimulation (rTMS) to suppress neural activity of the dPMC, S1, and primary motor cortex (M1) with single-pulse TMS over M1 for measuring cortico-spinal excitability (CSE) during kinesthetic and visual motor imagery of finger movements, as compared with static imagery conditions. They found that rTMS over both dPMC and S1 modulates the muscle-specific facilitation of CSE during kinesthetic but not during visual motor imagery. The stronger involvement of the dPMC and S1 in kMI than in vMI may explain the better outcomes attributed to rehabilitation programs for the improvement of motor functionality, focusing on the kinesthetic more than the visual strategy.

Classically, semantics refers to our capacity to attribute meaning to the events and entities (such as objects, words, feelings, and so on) that we experience during our lifespan and organize in a symbolic system. Language is the symbolic system that we use to represent this knowledge about the world. Current literature supports the notion that the recognition of objects, when visually presented, is subserved by neural structures different from those responsible for the semantic processing of their nouns. However, the embodiment approach foresees that processing observed objects and their verbal labels should share similar neural mechanisms. It is important to note that tools are a special class of graspable objects for humans since they have an associated functional use that involves a particular modality of interaction with the object, rather than just the feature to be grasped, as natural objects have. Functional neuroimaging studies show that tools are represented in circuits distinct from those where natural objects are represented. In a combined behavioral and MEG study, Visani et al. [6] compared the modulation of motor responses and cortical rhythms during the processing of graspable natural objects and tools, either verbally or pictorially presented. In line with the embodiment approach, findings demonstrated that conveying meaning to an observed object or processing its noun similarly modulates both motor responses and cortical rhythms; since natural graspable objects and tools are differently represented in the brain, they affect both behavioral and MEG findings in different manners, independent of presentation modality. The evidence that neural substrates responsible for conveying meaning to objects overlap with those where the object is represented supports an embodied view of semantic processing. In a review, Mazzuca et al. [7] extended this suggestion to abstract words. Concrete concepts refer to physical and perceivable entities in the world (e.g., hammer). Converging evidence has shown that concrete concepts are acquired earlier in life and are processed and remembered faster. Conversely, abstract concepts (e.g., justice), i.e., concepts referring to ideas or entities, have a general disadvantage in response times and are acquired later in life. In addition, while concrete concepts generally refer to things that can be experienced through the senses, and therefore can be indicated and manipulated, abstract concepts tend to be acquired mainly through linguistic inputs. The specific recruitment of linguistic information in the representation of abstract concepts has been confirmed by rating studies showing that abstract concepts are judged to be more associated with the mouth effector as compared with concrete concepts, which in turn are more associated with hands or other effectors eliciting action patterns. Neural evidence from TMS and fMRI studies has further elucidated the role of mouth motor areas in processing abstract meanings, in accordance with the viewpoint claiming that motor articulation is necessary for inner speech to occur.

Finally, two research articles suggested that the relationship between the sensorimotor system and cognitive functions is causal, showing that a specific sensorimotor training improves the related cognitive functions. Giachero et al. [8] reported a video-based action observation treatment (AO), which made use of a semi-immersive virtual reality (VR) environment, to investigate its therapeutic benefits in enhancing gardening skills in a group of participants with intellectual disabilities (IDs). Within the approach of embodied cognition, it is now a well-accepted notion that the observation of actions performed by others activates, in the perceiver, the same sensorimotor structures responsible for the actual execution of those same actions. This motor resonance relates to understanding, imitation learning, and predicting action outcomes. In rehabilitation programs for IDs, VR can provide a safe setting through which the users can practice skills which would be dangerous in the real world. Participants underwent fourteen weeks of training with two training sessions per week. In the first session, they were asked to carefully observe the VR video, where the correct procedure of the different stages for sowing zucchini was projected, while in the second one they looked at their previous recordings, in which the different stages of sowing were performed incorrectly. At the end of the fourteen weeks, each participant was again asked to perform the task without observing the virtual video. The results of the neuropsychological test and of the questionnaire administered to the caregivers and the independent raters clearly showed the positive impact of the treatment, indicating AO as an effective strategy for motor and cognitive enhancement in people with ID. In the same vein, Pancotti et al. [9] considered that embodied cognition theories suggest that observation of facial expression induces the same pattern of muscle activation, and that this contributes to emotion recognition. Therefore, it is proposed that the inability to form facial expressions affects emotional understanding. The authors assumed that physical training specifically developed to mobilize facial muscles could improve the ability to perform facial movements, and, consequently, spontaneous mimicry and facial expression recognition. To test this assumption, a group of patients with schizophrenia were recruited, typically showing a reduced ability to express and perceive facial emotions. At the beginning and at the end of the study, the experimental and control group were submitted to a facial expression categorization test and their data were compared. The experimental group underwent a training period, during which the lip muscles and the muscles around the eyes were mobilized through the execution of transitive actions. Participants were trained three times a week for five weeks. Results showed that the physical training improved the recognition of others’ facial emotions, specifically for the responses of “fear”, the emotion for which the recognition deficit in the test was most severe.

Overall, the contributions of this Special Issue provide novel data, new materials, and fruitful thoughts on the involvement of the sensorimotor system in cognitive functions. They are all in agreement with the viewpoint of embodied cognition which claims that the motor, sensory, and cognitive systems closely interact, and that cognitive processes are deeply rooted in the body’s interactions with the world. Hence, human cognition, rather than being centralized and sharply distinct from peripheral input and output modules, is closely related to sensorimotor processing. Furthermore, this relationship appears to be causal, as there is evidence that a specific deficit in the sensorimotor system results in a specific cognitive deficit.

## References

[1] Castellotti S., D’Agostino O., Biondi A., Pignatiello L., Del Viva M.M. (2022). Influence of Motor and Cognitive Tasks on Time Estimation. Brain Sci..

[2] Petrizzo I., Anobile G., Chelli E., Arrighi R., Burr D.C. (2022). Visual Duration but Not Numerosity Is Distorted While Running. Brain Sci..

[3] Craighero L., Marini M. (2021). Implicit associations between adverbs of place and actions in the physical and digital space. Brain Sci..

[4] Tosoni A., Altomare E.C., Brunetti M., Croce P., Zappasodi F., Committeri G. (2021). Sensory-motor modulations of eeg event-related potentials reflect walking-related macro-affordances. Brain Sci..

[5] Oldrati V., Finisguerra A., Avenanti A., Aglioti S.M., Urgesi C. (2021). Differential Influence of the Dorsal Premotor and Primary Somatosensory Cortex on Corticospinal Excitability during Kinesthetic and Visual Motor Imagery: A Low-Frequency Repetitive Transcranial Magnetic Stimulation Study. Brain Sci..

[6] Visani E., Sebastiano D.R., Duran D., Garofalo G., Magliocco F., Silipo F., Buccino G. (2022). The Semantics of Natural Objects and Tools in the Brain: A Combined Behavioral and MEG Study. Brain Sci..

[7] Mazzuca C., Fini C., Michalland A.H., Falcinelli I., Da Rold F., Tummolini L., Borghi A.M. (2021). From Affordances to Abstract Words: The Flexibility of Sensorimotor Grounding. Brain Sci..

[8] Giachero A., Quadrini A., Pisano F., Calati M., Rugiero C., Ferrero L., Pia L., Marangolo P. (2021). Procedural Learning through Action Observation: Preliminary Evidence from Virtual Gardening Activity in Intellectual Disability. Brain Sci..

[9] Pancotti F., Mele S., Callegari V., Bivi R., Saracino F., Craighero L. (2021). Efficacy of facial exercises in facial expression categorization in schizophrenia. Brain Sci..

